# HDAC6-dependent deacetylation of TAK1 enhances sIL-6R release to promote macrophage M2 polarization in colon cancer

**DOI:** 10.1038/s41419-022-05335-1

**Published:** 2022-10-21

**Authors:** Guangying Xu, Liling Niu, Youhui Wang, Guang Yang, Xingwu Zhu, Yuan Yao, Gang Zhao, Shaowei Wang, Hui Li

**Affiliations:** 1 Department of Gastrointestinal Cancer Biology, Tianjin Medical University Cancer Institute & Hospital, National Clinical Research Center for Cancer, Tianjin, China; 2 Key Laboratory of Cancer Immunology and Biotherapy, Tianjin, China

**Keywords:** Colon cancer, Post-translational modifications

## Abstract

Histone deacetylase 6 (HDAC6), a member of the HDAC family, has been identified as a potential therapeutic target for tumor therapy, but the function and underlying mechanisms of HDAC6 in colon cancer are incompletely characterized. Our study showed that the infiltration ratio of M2 macrophages was increased in colon cancer tissues with high HDAC6 expression. Similarly, the knockdown of HDAC6 in colon cancer cells inhibited cocultured macrophage M2 polarization in vitro. Analysis of the antibody chip revealed that HDAC6 promoted sIL-6R release to enhance macrophage M2 polarization. Mass spectrometry and immunoprecipitation demonstrated that, mechanistically, HDAC6 interacted with transforming growth factor β–activated kinase 1 (TAK1), deacetylated TAK1 at T178 and promoted TAK1 phosphorylation. TAK1-p38 MAPK signaling could further increase the phosphorylation and activity of ADAM17, which is responsible for shedding of IL-6R. Notably, the expression of phosphorylated TAK1 was positively correlated with HDAC6 expression and macrophage M2 polarization in human colon cancer tissues. Our study revealed a new HDAC6-TAK1-ADAM17 regulatory axis that mediates sIL-6R release and macrophage polarization in colon cancer.

## Introduction

Colon cancer is a common malignant tumor in the digestive system and is associated with high morbidity and mortality [1, 2]. Various complex factors in cancer cells and the tumor microenvironment (TME) are involved in the development of colon cancer [3]. Tumors affect immune regulation in a variety of ways to promote progression and metastasis [4]. Tumor-associated macrophages (TAMs) are some of the most abundant immune cells in the TME [5]. M2-type TAMs promote tumor growth and participate in tumor immunosuppression by stimulating angiogenesis and tissue remodeling [6]. The TAM infiltration level is high in colon cancer and is associated with a poor prognosis [7]. Therefore, more effective treatments targeted at immunosuppressive microenvironment induced by M2 macrophages in colon cancer need to be further explored.

The posttranslational modification of proteins plays an important role in tumor remodeling. Histone deacetylase 6 (HDAC6) is a deacetylase that participates in the dynamic regulation of the deacetylation of histone and nonhistone substrates [8]. It has been confirmed that the expression of HDAC6 is increased in a variety of tumor tissues and that HDAC6 can promote tumor development and metastasis [9, 10]. In melanoma models, HDAC6 inhibitors had a more significant inhibitory effect on tumor growth in C57BL/6 mice than in immunodeficient mice, which demonstrates that the antitumor effect of HDAC6 inhibitors requires an intact host immune system [9]. When HDAC6 inhibitors are combined with immune checkpoint inhibitors, they can effectively promote the release of IL-12 and TNF-α and polarize macrophages towards the antitumor M1 subtype [8, 9]. However, the mechanisms of HDAC6 in the colon cancer microenvironment, especially those underlying the effect on macrophage polarization, are still unclear. Interestingly, we found that HDAC6 could promote the release of sIL-6R by colon cancer cells, thereby further affecting macrophage M2 polarization.

Classical IL-6 signaling involves IL-6 ligation to membrane-bound IL-6 receptor (mIL-6R) and gp130 transmembrane receptor dimerization, whereas “IL-6 trans-signaling” is mediated by the soluble IL-6 receptor (sIL-6R), which forms a complex with IL-6 and directly engages gp130 [11, 12]. Unlike mIL-6R, which is expressed in only a few cells, the IL-6/sIL-6R complex can, in principle, be activated in all cells due to the universal expression of gp130 [13]. SIL-6R is produced either via the proteolysis of mIL-6R by the metalloprotease ADAM17 (A disintegrin and metalloproteinase-17) or less frequently, by the alternative splicing of IL-6R mRNA, which has only been detected in humans [13–15]. It has been confirmed that mIL-6R, as a source of sIL-6R, is also expressed in tumor cells [16, 17]. IL-6 trans-signaling not only promotes the malignant proliferation and metastasis of tumor cells but also affects the TME, as well as macrophage M2 polarization [18–20]. IL-6 trans-signaling upregulates the expression of the IL-4 receptor and enhances IL-4-induced STAT6 phosphorylation, which promotes the M2 polarization of macrophages [21]. Recent evidence has shown that sIL-6R expression is markedly increased during tumorigenesis, in addition, the expression of ADAM17 is increased in colorectal cancer cells compared with normal cells, which further confirms the effect of sIL-6R [13, 22]. Therefore, exploring the regulatory mechanism of sIL-6R release is of great significance for identifying clinical targets for colon cancer.

Transforming growth factor β-activated kinase 1 (TAK1) is a serine/threonine kinase and a member of the mitogen-activated protein kinase (MAPK) kinase kinase (MAP3K) family [23]. TAK1 is essential to produce tumor necrosis factor-α (TNF-α) and other cytokines via the activation of downstream MAPKs, such as p38 MAPK, extracellular signal-regulated kinases 1 and 2 (ERK1/2), and Jun N-terminal protein kinases 1 and 2 (JNK1 and JNK2) [24]. In addition, increasing evidence has demonstrated that the inhibition of TAK1 activity induces cancer cell death and prevents tumorigenesis and metastasis, suggesting that TAK1 may be an effective target in cancer treatment [25–27]. TAK1 kinase activity is regulated by a variety of posttranslational modifications. The phosphorylation of critical amino acid (aa) residues in the activation loop of TAK1 is critical for its kinase activity [28–30]. Besides phosphorylation, TAK1 can also undergo acetylation. It has been reported that YopJ acts as a serine/threonine acetyltransferase and prevents the phosphorylation of TAK1 by acetylating TAK1 at T184 and T187, inhibiting the activity of TAK1 [31]. However, research on the acetylation of TAK1 and the associated mechanism is relatively limited. Exploring the crosstalk among these modifications will provide a more comprehensive understanding of pathogenesis.

In this study, we investigated the role of HDAC6 in promoting macrophage M2 polarization in colon cancer. The knockdown of HDAC6 inhibited the release of sIL-6R in colon cancer cells, thereby inhibition M2-like macrophage polarization. Additionally, HDAC6 promoted TAK1 phosphorylation, activated p38 MAPK signaling and increased ADAM17 phosphorylation and activity. More importantly, we found that TAK1 is a novel substrate of HDAC6. HDAC6 interacts with TAK1 and deacetylates TAK1 at T178 to influence its kinase activity. Our findings reveal an underlying mechanism the HDAC6-mediated deacetylation of TAK1 regulation of sIL-6R release and suggest a promising strategy for inhibiting the development of colon cancer.

## Results

### Elevated HDAC6 expression in colon cancer cells promotes the M2 polarization of macrophages

**Fig. 1:**
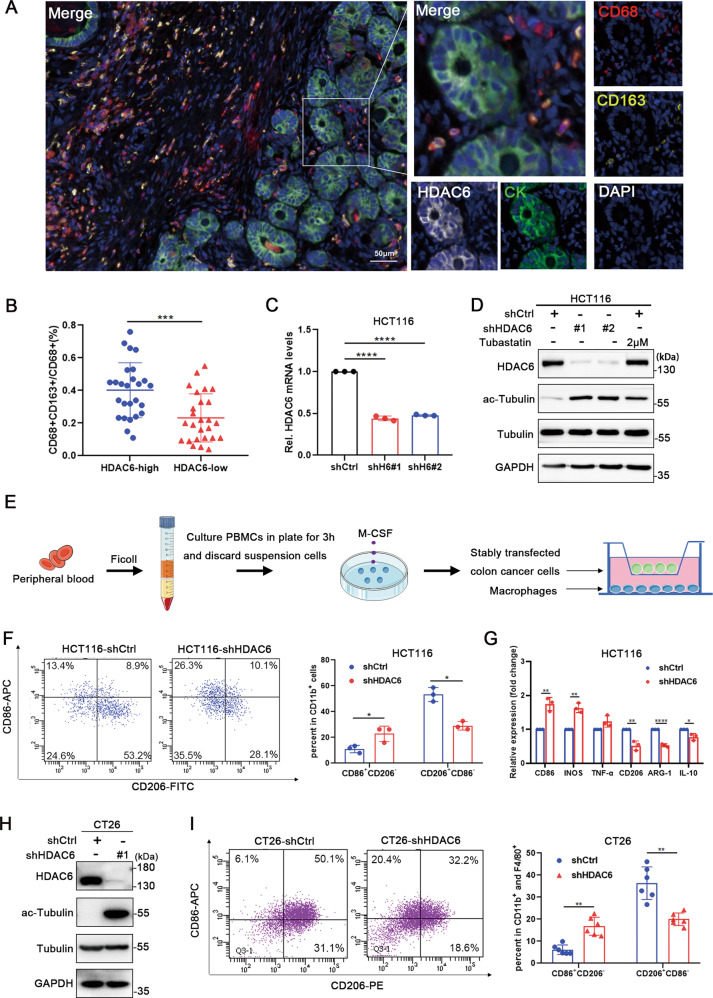
Elevated HDAC6 expression in colon cancer cells promotes the M2 polarization of macrophages. **A** HDAC6 protein expression in colon cancer and M2 macrophage infiltration in the microenvironment were evaluated using an mIHC platform (panel: CK/HDAC6/CD68/CD163). Representative mIHC images are shown, scale bar, 50 μm. **B** Fifty-two patients were divided into two groups according to HDAC6 expression using the median value as the cut-off, and then the proportion of CD68^+^CD163^+^/CD68^+^ cells were calculated. **C**, **D** Construction of HCT116 cell line with stable HDAC6 knockdown. Tubastatin A HCl is HDAC6 selective inhibitor and tubulin is the deacetylation substrate of HDAC6. **E** Schema of the construction of an in vitro model of stable HDAC6 knockdown colon cancer cells cocultured with macrophages derived from PBMCs. **F** Flow cytometry was used to assess the surface expression of CD11b, CD86 and CD206 in shCtrl- and shHDAC6-treated HCT116 cells. **G** QPCR detected expression of M1 and M2 polarization related genes of cocultured macrophages in shCtrl- and shHDAC6-treated HCT116 cells. **H** Construction of CT26 cell line with stable HDAC6 knockdown. **I** CT26-shCtrl or CT26-shHDAC6 cells suspension were subcutaneously injected into BALB/c mice (3 × 10^6^ cells per mouse, *n* = 12). After 22 days, flow cytometry was used to assess the surface markers of macrophage polarization in mouse tumor tissues. H6 is the abbreviation of HDAC6. Data were shown as the mean ± SD of three independent experiments, *P < 0.05; **P < 0.01; ***P < 0.001; ****P < 0.0001.

To evaluate the correlation between HDAC6 expression and macrophage M2 polarization in the colon cancer immune microenvironment, we first collected tissue samples from 52 patients with stage II-IV colon cancer for multiplex immunohistochemistry (mIHC) analysis (Fig. 1A). The median value was selected to separate specimens into groups with low and high HDAC6 expression. The data showed that HDAC6 expression was positively correlated with colon cancer metastasis and the tumor stage (Table 1), and that the proportion of infiltrating CD163^+^CD68^+^ macrophages was higher in the high-HDAC6 expression group (Fig. 1B), suggesting that the level of HDAC6 expression in tumor cells is related to the proportion of M2 macrophage infiltration. To further explore the effect of HDAC6 on macrophage polarization, we utilized an in vitro co-culture model of stable HDAC6-knockdown colon cancer cells and macrophages from healthy donors (Fig. 1C–E). HDAC6 knockdown in HCT116 cells significantly increased the proportion of CD86^+^CD206^-^ macrophages and decreased the proportion of CD86^-^CD206^+^ macrophages (Fig. 1F). QPCR results further showed the expression of M2-related genes (CD206, Arg-1, and IL-10) significantly decreased and M1-related genes (CD86, INOS) increased in macrophages when HDAC6 was knockdown in HCT116 cells (Fig. 1G). To detect the effect of HDAC6 on macrophage polarization in vivo, we subcutaneously injected CT26-shCtrl or CT26-shHDAC6 cells suspension into BALB/c mice (*n* = 12). After 22 days, the proportion of infiltrating M2 macrophages in mouse tumors was significantly decreased when HDAC6 was knocked down in CT26 cells (Fig. 1H–I). These results support the hypothesis that HDAC6 in colon cancer promotes macrophage polarization towards the M2-like phenotype.

**Table 1:** Relationship of HDAC6 expression with metastasis (*χ*2 test).

	HDAC6 expression		
Variable	Low	High	P value
Age	–	–	0.4022
≤60 years	13	16	–
>60 years	13	10	–
Gender	–	–	0.0479
Male	12	19	–
Female	14	7	–
Lymph node metastasis	–	–	0.012
Negative	19	10	–
Positive	7	16	–
Distant metastasis	–	–	0.0385
Negative	21	14	–
Positive	5	12	–
Stage	–	–	0.0006
I, II	16	4	–
III, IV	10	22	–

### HDAC6 promotes sIL-6R release in colon cancer cells by increasing the protease activity of ADAM17

**Fig. 2:**
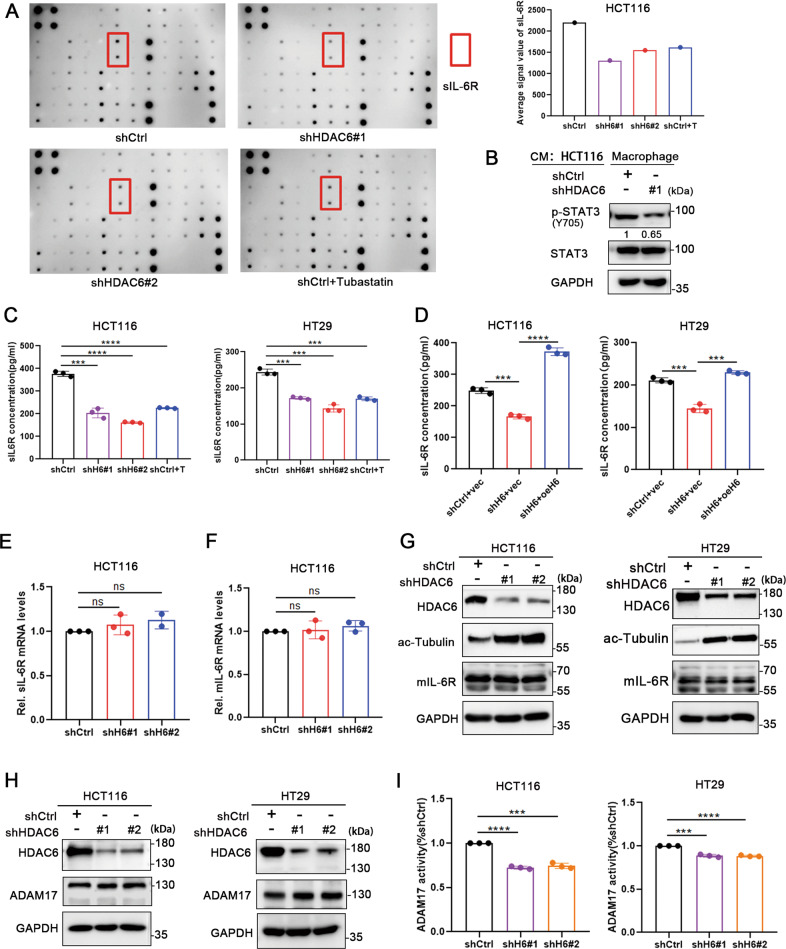
HDAC6 promotes sIL-6R release in colon cancer cells by increasing the activity of ADAM17. **A** Left, analysis of cytokine levels in the culture medium of HCT116 cells stably expressing shCtrl or shHDAC6 or treated with the HDAC6 inhibitor Tubastatin A HCl by using the AAH-INF-3 RayBiotech antibody chip. Right, the quantified average signal value of sIL-6R. **B** Western blot analysis of the total protein expression and phosphorylation level of STAT3 in macrophages cocultured with HCT116 cells. **C**, **D** SIL-6R contents in the culture medium of HCT116 and HT29 cells in the indicated groups determined by ELISA. **E**, **F** qRT-PCR analysis of the mRNA expression levels of mIL-6R and sIL-6R in HCT116 cells with stable HDAC6 knockdown. **G** Western blot analysis of mIL-6R protein levels in HCT116 and HT29 cells with stable knockdown of HDAC6. **H** Western blot analysis of ADAM17 protein levels in HCT116 and HT29 cells with stable HDAC6 knockdown. **I** Detection of the cleavage activity of ADAM17 (TACE Substrate IV, Fluorogenic) in **G**. AD17 is the abbreviation of ADAM17; H6 is the abbreviation of HDAC6; T is the abbreviation of Tubastatin A HCl. CM is the abbreviation of conditioned medium. Data were shown as the mean ± SD of three independent experiments; ns no significance, ***P < 0.001; ****P < 0.0001.

To determine the possible pathway by which HDAC6 affects macrophage polarization in the colon cancer microenvironment, we used a human inflammatory antibody array kit to analyze the culture medium of HCT116 cells exposed to different treatment conditions (Supplemental Fig. 1A). The results showed that sIL-6R contents in conditioned cell medium of HDAC6-knockdown cells or cells treated with a selective HDAC6 inhibitor, Tubastatin A HCl were significantly lower than that in the control group (Fig. 2A and Supplemental Fig. 1B). It has been reported that sIL-6R can activate IL-6/IL-6R/gp130 signaling pathway and promote M2 macrophage polarization [21, 32]. We firstly detected the phosphorylation of STAT3 in the macrophages cocultured with HCT116 cells. Compared to control cells, the phosphorylation of STAT3 in macrophages significantly decreased after cocultured with HDAC6 knockdown group (Fig. 2B). To validate the chip results, we measured the sIL-6R contents in cell culture medium collected from HCT116 and HT29 cells using ELISA. The results showed that the levels of sIL-6R were significantly decreased when HDAC6 was knocked down or inhibited by using selective inhibitor (Fig. 2C). In addition, the sIL-6R content was restored after HDAC6 overexpression (Fig. 2D). The above experimental results show that HDAC6 can promote sIL-6R release in colon cancer cells, which may be one of the reasons why HDAC6 promotes macrophage M2 polarization.

To further explore the pathway by which HDAC6 affects sIL-6R release, we subjected stable HDAC6-knockdown HCT116 cells to qRT-PCR analysis and found that HDAC6 knockdown did not affect the mRNA transcription levels of sIL-6R or mIL-6R (Fig. 2E, F). In addition, HDAC6 knockdown did not affect the protein expression of mIL-6R in colon cancer cells (Fig. 2G). Since the majority of sIL-6R is derived from proteolysis mediated by the metalloprotease ADAM17 [13–15], we knocked down ADAM17 with siRNA to verify its regulatory effect on sIL-6R release in colon cancer cells. The results confirmed that ADAM17 shed IL-6R in HCT116 cells (Supplemental Fig. 2A, B). Furthermore, knockdown of ADAM17 in HCT116 cells inhibited M2 polarization of cocultured macrophages (Supplemental Fig. 2C, D). To explore whether HDAC6 could affect ADAM17 expression and its activity, we measured the levels of the protein expression of ADAM17 and the cutting substrate peptides in colon cancer cells. The results showed that ADAM17 activity was significantly reduced after HDAC6 knockdown, while the protein expression of ADAM17 remains unchanged (Fig. 2H, I). Therefore, we confirmed that HDAC6 affects sIL-6R release in colon cancer cells by regulating the activity of ADAM17.

### HDAC6 promotes sIL-6R release by activating the TAK1-p38-ADAM17 axis

**Fig. 3:**
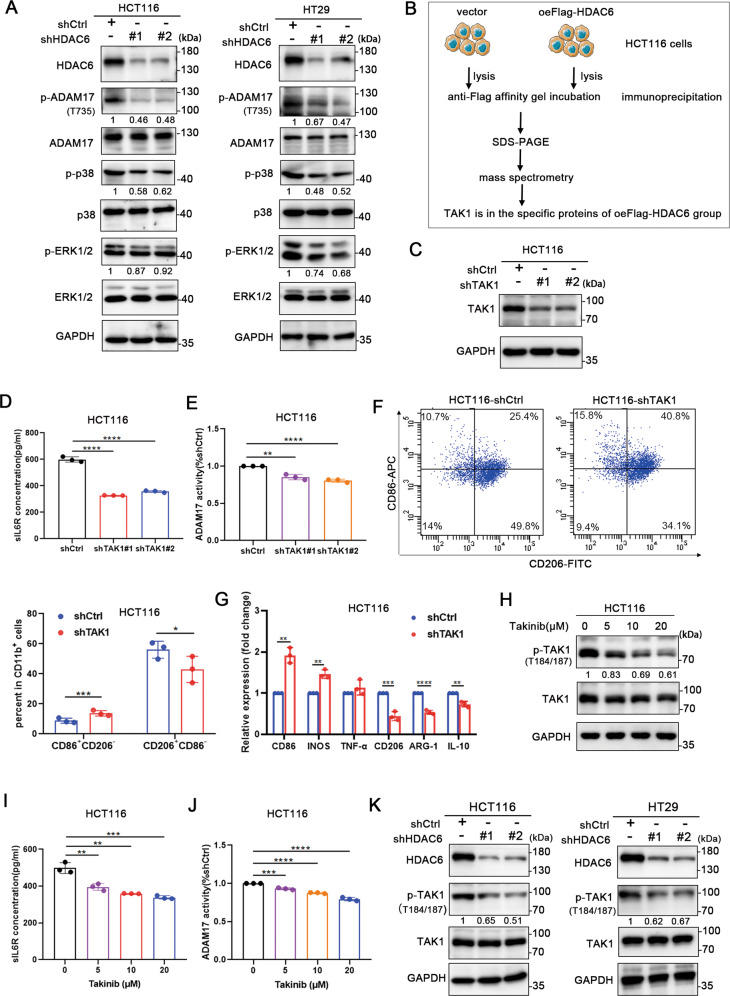
HDAC6 promotes sIL-6R release by activating the TAK1–p38–ADAM17 axis. **A** Western blot analysis of the total protein expression and phosphorylation of ADAM17, ERK1/2 and p38. GAPDH was used as an internal reference protein. **B** Schematic of the experimental procedure used to analysis of HDAC6 interacting proteins. The unique peptides and peptide-spectrum matches of TAK1 are 4, which is credible. **C**. Stable knockdown of TAK1 in HCT116 cells. **D**, **E** Analysis of sIL-6R levels in the cell media by ELISA **D** and detection of ADAM17 activity **E** in HCT116 cells after TAK1 knockdown. **F** Flow cytometry assessed CD11b, CD86 and CD206 expression of cocultured macrophages in shCtrl- and shTAK1-treated HCT116 cells. **G** QPCR detected the expression of M1 and M2 polarization related genes of cocultured macrophages in shCtrl- and shTAK1-treated HCT116 cells. **H** Treatment with the TAK1 selective inhibitor Takinib decreased TAK1 phosphorylation in HCT116 cells. **I**, **J** Analysis of sIL-6R levels in the supernatants by ELISA **I** and detection of ADAM17 activity **J** in HCT116 cells after treated with Takinib. **K** Western blot analysis of TAK1 total protein expression and phosphorylation in HCT116 and HT29 cells with stable HDAC6 knockdown. Data were shown as the mean ± SD of three independent experiments, *P < 0.05; **P < 0.01; ***P < 0.001; ****P < 0.0001.

The activity of ADAM17 is affected by many factors [33]. Phosphorylation of the threonine residue at position 735 (T735) in the intracellular domain has been confirmed to promote the protease activity of ADAM17, which is mediated by ERK1/2 and p38 MAPK [34, 35]. Therefore, the phosphorylation of ADAM17 (T735) was assessed in HDAC6 knockdown cells. The results showed that HDAC6 knockdown inhibited the phosphorylation of ADAM17 at T735 and upstream kinase (Fig. 3A). Thus, HDAC6 may regulate ADAM17 activity by affecting the intracellular phosphorylation of ADAM17 to promote the release of sIL-6R. However, previous studies showed no evidence that ADAM17 is a direct substrate of HDAC6. HDAC6 may indirectly regulate phosphorylation through interaction with any unknown kinases.

To further explore the mechanism more directly, protein mass spectrometry was used to detect proteins that can interact with HDAC6 in HCT116 cells. The mass spectrometry results did not show that HDAC6 interacts with p38, ERK, or ADAM17. However, the kinase TAK1, which is upstream of the MAPK signaling pathway, was specifically enriched in the HDAC6-overexpression group (Fig. 3B). To evaluate whether TAK1 was involved in the regulatory axis of sIL-6R release, we measured the ADAM17 activity and sIL-6R content when TAK1 was stably knocked down with shRNA as well as kinase activity inhibition by the selective inhibitor Takinib. As we expected, ADAM17 activity and sIL-6R secretion in HCT116 cells with TAK1 knockdown (Fig. 3C–E) or activity inhibition (Fig. 3H–J) were significantly reduced compared with those in control cells. Furthermore, knockdown of TAK1 in HCT116 cells inhibited M2 polarization of cocultured macrophages (Fig. 3F, G). To clarify whether HDAC6 is upstream of TAK1, we further examined the protein expression and phosphorylation of TAK1 in HCT116 and HT29 cells. Compared to control cells, shHDAC6-treated cells showed no significant difference in total TAK1 protein expression but exhibited a significant decrease in TAK1 phosphorylation (Fig. 3K). Therefore, HDAC6 promotes sIL-6R secretion possibly by regulating the kinase activity of TAK1 to activate its downstream signaling pathways.

### Inhibition of TAK1 activation blocks the HDAC6-induced activation of the TAK1-ADAM17 axis, release of sIL-6R and macrophage M2 polarization

**Fig. 4:**
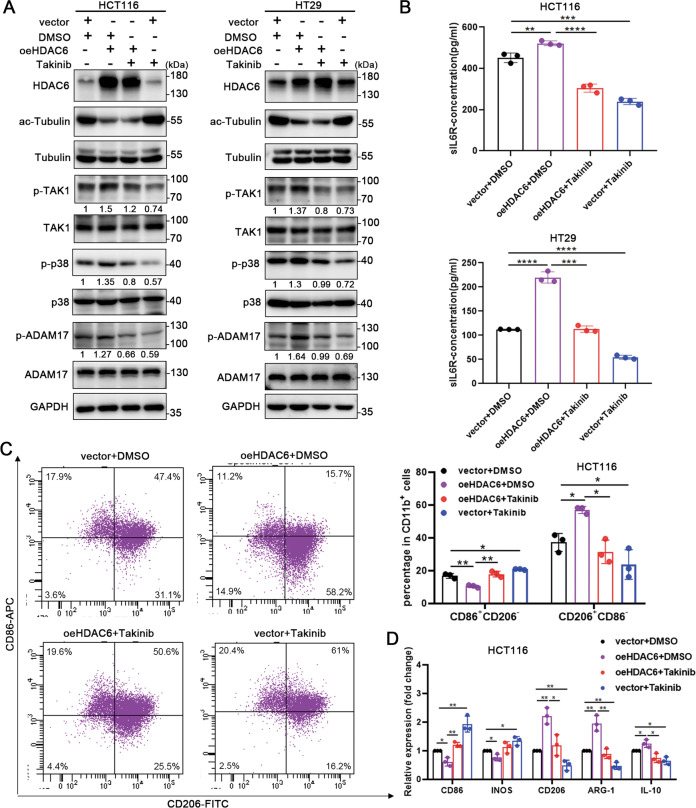
Inhibition of TAK1 activation blocks the HDAC6-induced activation of the TAK1–ADAM17 axis, release of sIL-6R and macrophage M2 polarization. **A** Western blot analysis of the phosphorylated and total protein expression of TAK1, ADAM17, and p38 in colon cancer cells treated with DMSO or Takinib (TAK1 selective inhibitor) after transfection with the empty vector or an HDAC6 overexpression plasmid. **B** Analysis of sIL-6R contents in the supernatants of HCT116 and HT29 cells in the indicated groups by ELISA. **C** Flow cytometry was used to evaluate the surface expression of CD11b, CD86, and CD206 of cocultured macrophages in the indicated groups. The statistical graphs (right) show the percentages of CD86^+^CD206^−^ and CD86^−^CD206^+^ cells among CD11b^+^ cells. **D** QPCR detected the expression of M1 and M2 polarization related genes of cocultured macrophages in the indicated groups. Data were shown as the mean ± SD of three independent experiments, *P < 0.05; **P < 0.01; ***P < 0.001; ****P < 0.0001.

We further verified whether HDAC6 affects downstream phenotypes by promoting TAK1 phosphorylation and activation. HDAC6 was firstly overexpressed in HCT116 or HT29 cells and then the cells were treated with the TAK1 selective inhibitor Takinib. The results indicated that the phosphorylation of TAK1, p38, and ADAM17 was significantly increased after HDAC6 overexpression and was restored to normal levels after treatment with Takinib (Fig. 4A). Similarly, the content of sIL-6R in the conditioned media of HCT116 cells increased when HDAC6 was overexpressed, and consistently decreased when treated with Takinib (Fig. 4B). Takinib could further inhibit the increase in the M2 polarization of macrophages induced by HDAC6 (Fig. 4C, D). In addition, the M2 polarization of macrophages was also reduced in the group treated with Takinib alone compared with the control group. In summary, these results show that the activation of TAK1 and downstream signal transduction play a critical role in the process, which HDAC6 promotes the release of sIL-6R and the polarization of M2 macrophages in colon cancer.

### HDAC6 interacts with TAK1

**Fig. 5:**
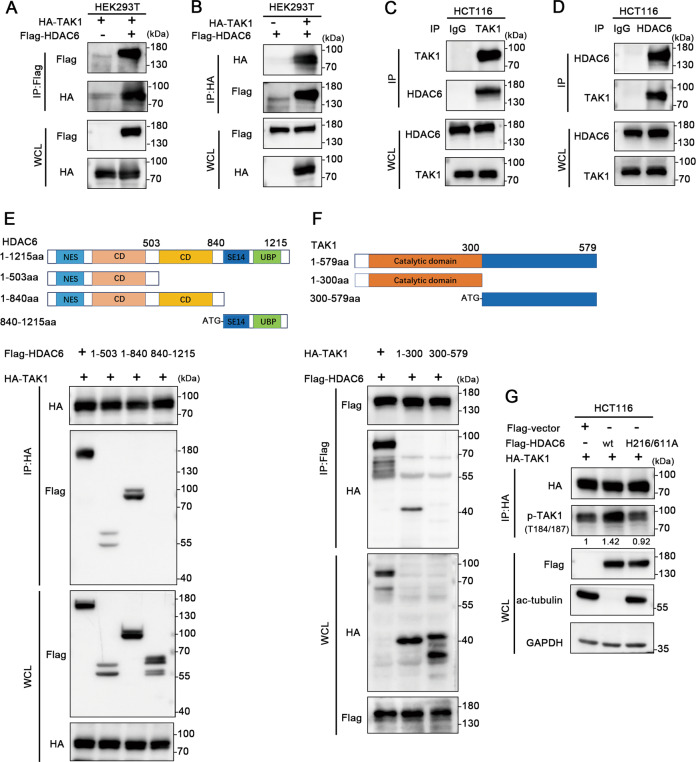
HDAC6 interacts with TAK1. **A**, **B** Interaction between HDAC6 and TAK1 was assessed by co-expressing the proteins in HEK293T cells. **C**, **D** Analysis of the endogenous interaction between TAK1 and HDAC6 in HCT116 cells by immunoprecipitation. **E**, **F** Binding domains between HDAC6 and TAK1 were determined using constructs expressing full-length and truncated HDAC6 and TAK1. **G**. Phosphorylation of exogenously expressed HA-TAK1 evaluated after transfection with the empty vector, Flag-HDAC6-WT or Flag-HDAC6-H216/611A. Whole-cell lysates were immunoprecipitated with an anti-HA antibody, and the precipitated proteins were probed with a p-TAK1(T184/187) antibody. WCL is the abbreviation of whole-cell lysate. WT is the abbreviation of wild type.

To figure out how HDAC6 influences TAK1 phosphorylation and activation, coimmunoprecipitation was primarily used to verify the interaction between HDAC6 and TAK1. Flag-tagged HDAC6 was co-expressed with HA-TAK1 in HEK293T cells, and a significant fraction of HA-TAK1 could be co-precipitated with an anti-Flag antibody (Fig. 5A). Flag-HDAC6 could also be pulled down by HA-TAK1 (Fig. 5B). Further, reversible coimmunoprecipitation confirmed the interaction between endogenous expressed TAK1 and HDAC6 (Fig. 5C, D). In addition, to identify the definitive binding domain between HDAC6 and TAK1, we used a series of truncated fragments of the two proteins and found that the 1-840 aa region of HDAC6, which contains two deacetylase activity domains, binds full-length TAK1. Similarly, the 1-300 aa region of TAK1, which includes the kinase domain, bound full-length HDAC6 (Fig. 5E, F). These interaction domains provided evidence that HDAC6 may regulate TAK1 activation depending on its deacetylase domain. In HCT116 cells, HA-tagged TAK1 was coexpressed with HDAC6-Flag and HDAC6 deacetylase mutants (HDAC6-H216/611A-Flag) [36]. We found that the level of phosphorylated TAK1 was significantly increased after HDAC6 overexpression, while TAK1 phosphorylation did not change in the group expressing the HDAC6 deacetylase mutants, which have lacked deacetylation ability, compared with the control group (Fig. 5G). Taken together, these results suggest that HDAC6 may regulate the kinase activity of TAK1 through deacetylation TAK1.

### HDAC6 promotes TAK1 phosphorylation by deacetylating it at T178

**Fig. 6:**
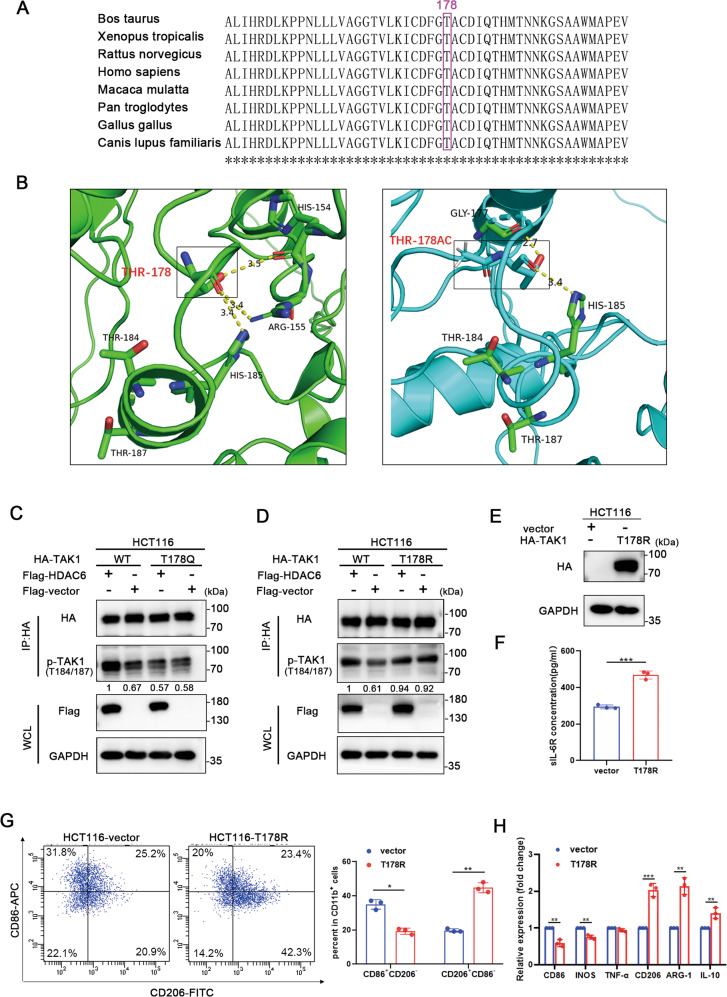
HDAC6 upregulates TAK1 phosphorylation by deacetylating TAK1 at T178. **A** T178 in TAK1 is evolutionarily conserved. **B** Acetylation of the T178 residue of TAK1 as mimicked (left, wild type TAK1; right, T178Ac TAK1). The related sites are marked in the picture. **C**, **D** HA-tagged TAK1 was coexpressed with the empty vector or Flag-HDAC6, and an HA-tagged TAK1 acetylation mimic mutant (left, T178Q) or deacetylation mimic mutant (right, T178R) was coexpressed with the empty vector or Flag-HDAC6. After the transfection of HCT116 cells, total cell lysates were immunoprecipitated with an anti-HA antibody, and the precipitated proteins were probed with a p-TAK1 (T184/187) antibody. **E** HA-tagged TAK1-T178R or vector was overexpressed in HCT116 cells. **F** Analysis of sIL-6R levels in the cell media by ELISA. **G** Flow cytometry assessed the surface expression of CD11b, CD86 and CD206 of cocultured macrophages in the indicated groups. **H** QPCR detected the expression of M1 and M2 polarization related genes of cocultured macrophages in the indicated groups. Data were shown as the mean ± SD of three independent experiments, *P < 0.05; **P < 0.01; ***P < 0.001.

Previous studies have shown that the acetyltransferase YopJ can catalyze the acetylation of serine and threonine residues in the kinase domain of TAK1, thereby preventing the phosphorylation of these sites and activating the downstream signaling pathways [31]. In addition, the analysis of posttranslational modifications by mass spectrometry revealed that lysine residues in TAK1 were acetylated, but the effect of lysine acetylation on the function of TAK1 has not been studied [31]. Since the acetylation of lysine residues is more common and easier to detect, we first tested whether lysine residues in TAK1 can be acetylated. Unexpectedly, the endogenous immunoprecipitation results showed that the acetylation of lysine residues in TAK1 was almost undetectable, indicating that HDAC6 may not regulate lysine acetylation of TAK1 (Supplemental Fig. 3A). To identify the acetylated functional sites of TAK1, we immunoprecipitated endogenous TAK1 from HCT116 cells and performed mass spectrometry assay. The mass spectrometric analysis suggested the acetylation site of TAK1 is T178 (threonine 178), which locates in the kinase domain. Sequence alignments in different species revealed that that T178 is a highly evolutionarily conserved residue, indicating the potentially critical role for T178 in the function of TAK1 (Fig. 6A).

It has been reported that the phosphorylation of T178, T184, and T187 residues of TAK1 is important for TAK1-mediated signal transduction [28, 37, 38]. Since the T184 and T187 residues are the key sites of TAK1 activation, these two sites were used to detect the phosphorylation of TAK1. We simulated the acetylation of TAK1 at T178 to preliminarily predict the corresponding effect on TAK1 activation. The AlphaFold model was performed to predict the protein structure of TAK1, and it was found that the addition of an acetyl group on the T178 residue of TAK1 could change the spatial conformations of the T184 and T187 sites. Moreover, spatially, the three sites are relatively close, and the acetylation of T178 may affect the phosphorylation modification of the other two sites or their interaction with other molecules, further affecting the phosphorylation and activation of TAK1 (Fig. 6B). To further examine the associated effects, we mutated the T178 residue to a glutamine residue (to mimic acetylation) (T178Q) or an arginine residue (to mimic deacetylation) (T178R). The results showed that when the T178 residue of TAK1 was mutated, the HDAC6-mediated regulation of TAK1 phosphorylation was abolished, indicating that HDAC6 exerts its effect by acting on the T178 residue. In addition, in the absence of HDAC6, the phosphorylation of the T178R mutant was increased, while that of the T178Q mutant was decreased, showing that deacetylation at T178 promotes the phosphorylation of TAK1 (Fig. 6C, D). To further elucidate the function of deacetylation of T178, we overexpressed vector or TAK1-T178R to mimic TAK1 deacetylation in HCT116 cells (Fig. 6E). In this situation, HCT116 cells with overexpression of TAK1-T178R released more sIL-6R than the vector group (Fig. 6F) and promoted polarization of cocultured macrophages to M2 subtype (Fig. 6G, H). Collectively, the above results indicate that HDAC6-mediated T178 deacetylation positively regulates TAK1 phosphorylation.

### Phosphorylation of TAK1 is positively correlated with HDAC6 expression in colon cancer

**Fig. 7:**
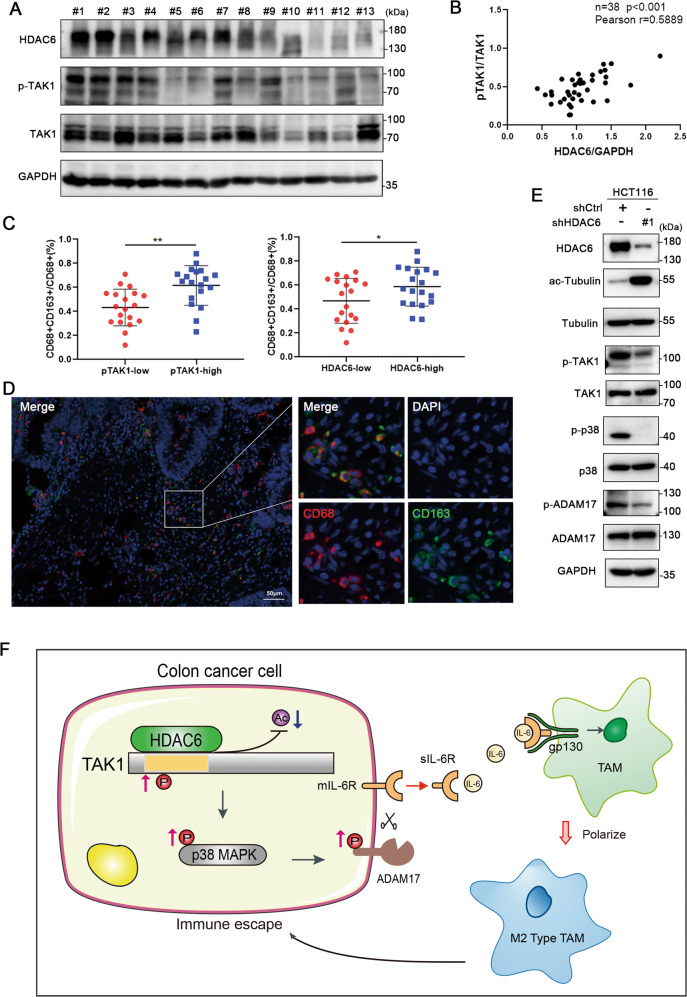
TAK1 phosphorylation is positively correlated with HDAC6 expression in colon cancer. **A** Protein was extracted from 38 fresh postoperative colon cancer tissues for western blot analysis of p-TAK1, TAK1, and HDAC6 protein levels. Relative protein levels were normalized by GAPDH. Thirteen samples are shown. **B** Quantification of the intensities of the indicated proteins was performed using ImageJ software, followed by Pearson’s correlation coefficient analysis. **C**, **D** M2 macrophage infiltration in the 38 colon cancer tissues was evaluated using an mIHC platform (panel: CD68/CD163). Patients were divided into two groups according to p-TAK1/TAK1 or HDAC6/GAPDH expression based on western blot analyses of tissue proteins, and then the proportion of CD163^+^CD68^+^/CD68^+^ cells were calculated. Representative mIHC images are shown, scale bar, 50 μm. **E** Western blot analysis of the phosphorylated and total protein expression of TAK1, ADAM17, and p38 in xenograft mouse models (BALB/c nude) originating from HCT116-shCtrl and HCT116-shHDAC6 cells. **F** Working model. WCL is the abbreviation of whole-cell lysate. Data were shown as the mean ± SD, *P < 0.05; **P < 0.01.

Our findings demonstrate that HDAC6 promotes the phosphorylation and activation of TAK1 by deacetylating it in colon cancer cells. Thus, we investigated the protein levels of phosphorylated TAK1, TAK1, and HDAC6 in human colon cancer. We performed a western blot analysis of 38 fresh postoperative colon cancer tissue specimens (Fig. 7A and Supplemental Fig. 4A). In these 38 samples, HDAC6 expression was significantly positively associated with phosphorylated TAK1 (*r* = 0.5889, *p* < 0.001; Fig. 7B). Moreover, the proportion of infiltrating CD163^+^CD68^+^ macrophages was higher in the high-phosphorylated TAK1 expression group and high-HDAC6 expression group (Fig. 7C, D). These clinical data indicate that TAK1 phosphorylation is positively correlated with HDAC6 expression and is related to the proportion of M2 macrophage infiltration in colon cancer specimens. To further verify the regulatory axis in vivo, we detected the critical nodes in tumor tissues of xenograft mouse model originating from HCT116-shCtrl and HCT116-shHDAC6 cells by western blot. The results showed that knockdown of HDAC6 inhibited phosphorylation of TAK1, p38, and ADAM17 in colon cancer xenograft mouse model, which were consistent with results in vitro (Fig. 7E). Therefore, the phosphorylation of TAK1 and HDAC6 may be potential biomarkers for human colon cancers.

## Discussion

In our study, we identified HDAC6 as a negative modulator of the immune microenvironment in colon cancer. Clinical data showed that elevated HDAC6 expression was associated with M2 macrophages infiltration. Further analysis demonstrated that HDAC6 in colon cancer cells promoted the polarization of M2 macrophages by regulating sIL-6R release. Mechanistically, HDAC6 interacts with TAK1 and TAK1 is a novel target of HDAC6. The HDAC6-mediated deacetylation of TAK1 at T178 increased the activation of the TAK1-p38 MAPK signaling and ADAM17 phosphorylation, thereby increasing the cleavage activity of ADAM17 and generating elevated sIL-6R (Fig. 7F). We found that phosphorylated TAK1 is positively correlated with HDAC6 and infiltration ratio of M2 macrophages in colon cancer tissues. Therefore, HDAC6 represents a promising therapeutic target related to the TME in colon cancer.

In the past few decades, HDAC6 has been recognized as an important member of the HDAC family because it contains two deacetylase catalytic domains and is mainly located in the cytoplasm [39, 40]. HDAC6 targets a wide range of substrates and participates in various biological functions, especially tumorigenesis [41]. However, the substrates and mechanisms reported thus far are insufficient to explain the broad functions of HDAC6. Here, our findings identified TAK1 as a novel target of HDAC6. TAK1, as a key regulator of activation signaling pathways such as the MAPK pathway, plays a role in transmitting upstream signals from receptor complexes to downstream signals [23, 42]. A substantial amount of evidence has indicated that posttranslational modifications, especially phosphorylation, play a critical role in properly controlling TAK1 activation according to the cellular environment [28]. Recent studies have revealed that HDAC6 overexpression can activate the ERK/p38 MAPK signaling pathway and promote the release of proinflammatory cytokines such as TNF-α into the inflammatory environment [43, 44]. The inhibition of TAK1, p38 MAPK, and HDAC6 can block the downregulation of Sig1R mRNA expression in microglia, however, the regulatory relationship between TAK1 and HDAC6 is unclear [45]. To the best of our knowledge, this is the first study showing that HDAC6 interacts with TAK1 and promotes its phosphorylation and the activation of downstream signaling pathways. Moreover, we discovered that HDAC6 deacetylates TAK1 at T178. It has been reported that three conserved threonine residues in the kinase activation loop of TAK1, T184, T187, and T178 are essential for TAK1 autophosphorylation and downstream signaling cascade activation and that the phosphorylation of T184 and T187 is more common [28, 37, 38]. Therefore, Thr184 and Thr187 can be used to reliably evaluate the phosphorylation of TAK1 and predict the function of T178 acetylation. Simulations of TAK1 T178 acetylation and T178 mutants were used to emphasize the effect of the deacetylation of TAK1 at T178 on regulating TAK1 phosphorylation and activation. However, our results do not explain how HDAC6 deacetylates TAK1 at T178. The main obstacle is the absence of antibodies that can detect threonine acetylation. Nonetheless, our findings are innovative and provide a new possibility, which HDAC6 can also deacetylate threonine. It has been reported that after YopJ acetylates threonine residues in TAK1, phosphorylation can no longer occur, resulting in the inhibition of kinase activity [31, 46]. We assume that HDAC6 deacetylates T178 of TAK1, thereby promoting the phosphorylation of this site or affecting the phosphorylation of T184 and T187, ultimately activating TAK1. However, the crosstalk between phosphorylation and acetylation is complicated, and further research is needed. Therefore, this finding expands the deacetylation substrates of HDAC6 and provides a new perspective on the mechanism of HDAC6 acetylation modification.

It has been reported that high HDAC6 expression indicates a poor colon cancer prognosis and can affect the proliferation and motility of colon cancer cells [47]. In addition to affecting the biological behavior of the tumor itself, HDAC6 also plays a role in regulating the TME. For example, HDAC6 inhibitors can inhibit the immunosuppressive function of Treg cells in lung cancer [48]. Moreover, HDAC6 inhibitors improve the therapeutic efficacy of anti-PD-1 immunotherapy by reducing the number of anti-inflammatory M1 macrophages and downregulating the expression of immune checkpoints such as PD-L1 in tumor cells [9]. Therefore, exploring the downstream targets of HDAC6 will help reveal the associated mechanisms and improve its utilization as a target in clinical practice. In our study, we determined that HDAC6 could promote sIL-6R release by colon cancer cells to regulate macrophage polarization. After sIL-6R is released, on the one hand, it activates the JAK/STAT3 pathway or other signaling pathways in the tumor itself in an autocrine manner and promotes the proliferation and metastasis of colon cancer cells. On the other hand, sIL-6R releases into the TME can combine with IL-6 to regulate other cells. Particularly, sIL-6R-mediated trans-signaling has been observed to result in the recruitment of macrophages for chemotaxis and infiltration in obesity models [49]. Research has indicated that MCT-1 regulates sIL-6R release in breast cancer cells, and the coculture of MCT-1-overexpressing breast cancer cells with THP-1 cells showed that MCT-1 promotes macrophage M2 polarization. Besides, an IL-6R inhibitor antagonized the effect of MCT-1 on macrophage polarization [32]. Moreover, M2 macrophages can in turn promote tumor progression by stimulating tumor angiogenesis and inhibiting T-cell function [50]. Notably, we found that HDAC6 knockdown inhibits ADAM17 activity. One important function of ADAM17 is the shedding of IL-6R, which is a prerequisite for IL-6 trans-signaling [15]. It has been reported that the absence of ADAM17 is sufficient to inhibit IL-6 trans-signaling-mediated tumor formation in the ApcMin/+ model [18]. These studies also provide support for the existence of a HDAC6-ADAM17-sIL-6R regulatory axis in colon cancer.

In summary, our study identified TAK1 as a novel substrate of HDAC6. HDAC6-mediated T178 deacetylation of TAK1 positively regulates TAK1 phosphorylation and activates the downstream signaling pathway. Furthermore, HDAC6 enhances ADAM17 activity, increases the release of sIL-6R and promotes the M2 polarization of macrophages in the TME, thus allowing immune escape and promoting tumor development. This study enhances our understanding of the functions and mechanisms of HDAC6 in human colon cancer immune microenvironment and provides a series of potential novel therapeutic targets for colon cancer treatment.

## Materials and methods

### Tumor samples

All colon cancer samples were obtained from the Tianjin Cancer Hospital. Pathological diagnosis and staging of colon cancer were performed according to the American Joint Committee on Cancer (AJCC) Staging System. The patient data and samples were collected with approval by the Ethics Committee of the Tianjin Cancer Hospital. Informed consent was obtained in all cases.

### Cell culture and treatment

Macrophages were derived from PBMCs and stimulated with 25 ng/ mL recombinant human M-CSF (PeproTech, USA, 300-25) for 6 days. The human colon cancer cell lines HCT116, HT29, and HEK293T cell lines were obtained from ATCC. All human cell lines had been authenticated using STR profiling (Tianjin genink biotechnology Co., Ltd) within the last 3 years. All experiments were performed with mycoplasma-free cells. HCT116 cells were cultured in McCoy’s 5A medium (Gibco, USA). HEK293T and HT29 cells were cultured in DMEM (Gibco). Macrophages were cultured in RPMI 1640 (Gibco). All media were supplemented with 10% fetal bovine serum (Gibco) and 1% penicillin/streptomycin (Gibco). The cells were maintained at 37 °C in a humidified atmosphere of 5% CO_2_. For transient transfection, the cells were transfected with plasmid or siRNA (Genechem, Shanghai, China) using Lipofectamine 3000 and Opti-MEM (Invitrogen). The ratio of Lipofectamine 3000 (µl) to: total DNA (μg) was 2:1, according to the manufacturer’s protocol. The sequences of siRNAs targeting ADAM17 were as follows (5′-3′): si1: CCTATGTCGATGCTGAACAAA; si2: CCCATGAAGAACACGTGTAAA. Lentiviruses carrying short hairpin RNA sequences targeting human HDAC6 or TAK1 mRNA (shHDAC6 or shTAK1, respectively) and matched negative controls were constructed by our laboratory. The sequences of shRNAs targeting human HDAC6 and TAK1 are as follows (5′-3′):

shHDAC6#1: GCATCCCATCCTGAATATCCTT

shHDAC6#2: GCATCCCATCCTGAATATCCTT

shTAK1#1: CGGAACCTTTAGGGATAGTTC

shTAK1#2: GCAGTGATTCTTGGATTGTTT

### Fluorescent Multiplex Immunohistochemistry

Paraffin sections of colon cancer tissues were collected and used for mIHC with an Opal 7-colour kit (PerkinElmer, NEL811001KT). For the first and subsequent rounds antigen epitope retrieval was performed in EDTA (pH 8.0) buffer using a microwave at the lowest power for 15 min. The slides were cooled to room temperature, and washed with TBST (three times, 3 min). Then, the slides were blocked with antibody diluent/block buffer for 10 min and incubated with primary antibody at 4 °C overnight. The following primary antibodies were used: anti-pan-CK (Abcam, Cambridge, UK, ab27988,); anti-HDAC6 (Cell Signaling Technology, USA, 7558); anti-CD68(Invitrogen, CA, USA, 14-0688-82); and anti-CD163(Abcam, ab182422). The next day, the slides were placed at room temperature and incubated with HRP-conjugated secondary antibody for 10 min after being washed with TBST three times. The slides were treated with TSA dye for 10 min, and then microwaved for epitope retrieval to label with the next antibody. Finally, the nuclei were stained with DAPI for 10 min at room temperature.

### Western blot analysis

Total protein was isolated from human colon cancer samples or cultured cells using precooled cell lysis buffer (containing 50 mM Tris-HCl pH 7.5, 150 mM NaCl, and 1% Triton) supplemented with protease inhibitor cocktail (MCE, NJ, USA, HY-K0010), deacetylase inhibitor cocktail (MCE, HY-K0030) and phosphatase inhibitor cocktail (MCE HY-K0022 and HY-K0023). The cell lysate was centrifuged at 4 °C and then the supernatant was collected. The protein concentration was determined using a BCA Protein Assay Kit (Thermo Fisher Scientific, MA, USA). A proper amount of protein was added to SDS loading buffer and boiled for 5 min. Then, the protein samples were separated by SDS/PAGE and transferred onto polyvinylidene difluoride membranes (Millipore). The membranes were blocked for 1 h with Tris-buffered saline with 0.1% Tween 20 containing 5% non-fat milk. For phosphorylation analysis, 5% bovine serum albumin was used. Antibody diluent (TOYOBO, Japan, NKB-101) was used to prepare primary and secondary antibodies. After incubation with primary antibodies overnight at 4°C, the membranes were washed and incubated with HRP-conjugated anti- rabbit (CST, 7074) or anti- mouse (absin, Shanghai, China) secondary antibodies. The protein bands were detected using ECL substrate (Millipore, WBKLS0500), visualized using an exposure instrument (Amersham Imager 600) and quantified using ImageJ software.

### Immunoprecipitation

HEK293T cells and HCT116 cells were transfected with the indicated plasmids for 48 h and then lysed. The samples were centrifuged, and the supernatant was collected. For each sample, 1 mg of protein was incubated overnight at 4 °C with 2 μg of the indicated antibody. The next day, protein-A/G beads (Bimake, TX, USA, b23202) or protein A/G agarose (Santa Cruz, CA, USA, sc-2003) was added for another 3 h at 4 °C. After being washed three times with cold IP buffer, the proteins were boiled with 2× SDS loading buffer for 10 min. The samples were centrifuged and used for follow-up experiments.

### Collection of conditioned medium

To obtain conditioned medium (CM), cells were grown to sub confluence. The culture medium was replaced with 1% FBS. After 24 h, the medium was harvested, and debris was removed by centrifugation at 1000×*g* for 10 min at 4 °C.

### ELISA

The conditioned medium of tumor cells was analyzed by ELISA using a commercial kit, according to the manufacturer’s protocol (human IL-6 receptor ELISA kit, Abcam, 46029).

### Determination of TACE/ADAM17 activity

When the cell density reached approximately 80%, the cells were detached and lysed in buffer (150 mM NaCl,50 mM Tris,1% Triton X-100, and 4% glycerol) supplemented with protease inhibitor cocktail (MCE, HY-K0010) and phosphatase inhibitor (MCE, HY-K0021 and HY-K0023) for 0.5 h at 4 °C. The proteins were centrifuged at 4 °C for 10 min at maximum speed, and then the protein concentration was determined using the BCA assay (Thermo Fisher Scientific, 23227). 20 μg protein was transferred to each well of a black 96-well plate. Then 20 μM TACE substrate IV (Merck Millipore, GER, 616407) was added and the samples were incubated for 30 min at 37 °C. ADAM17 activity was measured with a spectrophotometer at an excitation wavelength of 320 nm and an emission wavelength of 420 nm.

### Plasmid constructs

Full-length HDAC6, and TAK1 and the truncated fragments of both genes were amplified by PCR and cloned into the pcDNA3.1(+)-Flag and pcDNA3.1(+)-HA vectors. Point mutations of the indicated TAK1 constructs were purchased from Tsingke Biotechnology, Beijing.

### Flow cytometry

Macrophages from PBMCs were cocultured with stably transfected colon cancer cells or conditioned medium for 48 h.Then these macrophages were digested with cell dissociation buffer (Gibco, USA) and dissociated into single cell suspensions. Next, the cells were incubated with APC-conjugated anti-human CD11b (Biolegend,101228); PE/Cy5.5-conjugated anti-human CD86 (Biolegend, CA, USA, 305412) and FITC-conjugated anti-human CD206 (Biolegend, 321104) antibodies for 20 mins at 4 °C in the dark. Flow cytometry was performed using a flow cytometer (BD Biosciences, FACSCanto II, USA).

### RNA extraction and quantitative PCR

Total RNA was isolated from cultured cells using TRIzol reagent (Invitrogen, CA, USA). The mRNA was reverse transcribed into cDNA using PrimeScript RT Master Mix (TaKaRa, RR036A, Japan). TB Green Premix Ex Taq^TM^ II (TaKaRa, RR820A, Japan) was used to detect the amplified PCR products. Real-time PCR was performed using an ABI QuanStudio5 real time PCR instrument. The data were calculated using the 2^-ΔΔCT^ method and GAPDH was used as the reference. The primers used for different target genes are as follows:

HDAC6 (forward): 5ʹ-GAGGGAGAACTCCGTGTCCTA-3ʹ

HDAC6 (reverse): 5ʹ-AATGCCATCCATAAGACTGTGC-3ʹ

GAPDH (forward): 5ʹ-ACAACTTTGGTATCGTGGAAGG-3ʹ

GAPDH (reverse): 5ʹ-GCCATCACGCCACAGTTTC-3ʹ

sIL-6R (forward): 5ʹ-GCGACAAGCCTCCCAGGTTC-3ʹ

sIL-6R (reverse): 5ʹ-GTGCCACCCAGCCAGCTATC-3ʹ

mIL-6R (forward): 5ʹ-CATTGCCATTGTTCTGAGGTTC-3ʹ

mIL-6R (reverse): 5ʹ-GTGCCACCCAGCCAGCTATC-3ʹ

CD206 (forward): 5ʹ-GGGTTGCTATCACTCTCTATGC-3ʹ

CD206 (reverse): 5ʹ-TTTCTTGTCTGTTGCCGTAGTT-3ʹ

CD86 (forward): 5ʹ-CTGCTCATCTATACACGGTTACC-3ʹ

CD86 (reverse): 5ʹ-GGAAACGTCGTACAGTTCTGTG-3ʹ

ARG-1 (forward): 5ʹ-TGGACAGACTAGGAATTGGCA-3ʹ

ARG-1 (reverse): 5ʹ-CCAGTCCGTCAACATCAAAACT-3ʹ

INOS (forward): 5ʹ-GTTCCAGATGAATACTGGCAGTC-3ʹ

INOS (reverse): 5ʹ-GCAACTGAACACTATCTTTCCCT-3ʹ

TNF-α (forward): 5ʹ-GAGGCCAAGCCCTGGTATG-3ʹ

TNF-α (reverse): 5ʹ-CGGGCCGATTGATCTCAGC-3ʹ

IL-10 (forward): 5ʹ-CCTCCGTCTGTGTGGTTTGAA-3ʹ

IL-10 (reverse): 5ʹ-CACTGCGGTAAGGTCATAGGA-3ʹ

### Animal models

Mice were purchased from the Vital River Laboratory Animal Technology Co., Ltd (Beijing, China) and sheltered under specific pathogen-free conditions. CT26-shCtrl and CT26-shHDAC6 cells were suspended in PBS and subcutaneously injected into BALB/c mice (female, 5–6 weeks old, 3 × 10^6^ cells per mouse, *n* = 12). After 22 days, the mice were euthanized, and tumor tissues were removed for follow-up experiments. For the xenograft model, HCT116-shCtrl and HCT116-shHDAC6 cells were subcutaneously injected into BALB/c nude mice (female, 5–6 weeks old, 3 × 10^6^ cells per mouse, *n* = 10). All mice were randomly allocated. The experiment was conducted according to the ethics guidelines for animal experiments and approved by the Animal Ethical and Welfare Committee of Tianjin Medical University Cancer Institute and Hospital.

### Antibodies and reagents

Selective HDAC6 inhibitor, Tubastatin A HCl (HY-13271) was purchased from MCE and selective TAK1 inhibitor, Takinib (s8663) was purchased from Selleck. Anti- pan-Acetylated lysine (9441), anti-TAK1 (5206), anti-p-TAK1 (4508), anti-HDAC6 (7558), anti-HDAC6 (7558), anti-Flag (14793), anti-ERK1/2 (4695), anti-p-ERK1/2 (4377), anti-ADAM17 (3976), anti- p38 (8690), anti-p-p38 (4511), anti-IgG (2729), anti-Ac-tubulin (5335), anti-Tubulin (2148), anti-STAT3 (12640), and anti-p-STAT3 (9145) antibodies were obtained from Cell Signaling Technology. Anti-IL-6R (sc-373708) antibody was purchased from Santa Cruz Biotechnology. Anti-p-ADAM17 (ab182630) antibody was obtained from Abcam. Antibodies were used at a ratio of 1:1000 dilution. Anti-GAPDH (G8795) antibody was from Sigma-Aldrich and was used at a ratio of 1:3000 dilution.

### Statistical analysis

All statistical analyses were performed using GraphPad Prism 8.0 software and Microsoft Excel 2017 statistical program. Pearson’s correlation coefficient and the chi-square test were used to analyze associations between HDAC6 expression and the characteristics of patients with colon cancer. Statistical analyses between groups were performed by two-tailed Student’s *t* test. All data are expressed as the mean ± SD of three independent experiments. *P* values < 0.05 were considered statistically significant. *P < 0.05; **P < 0.01; ***P < 0.001; ****P < 0.0001; ns=not significant.

## Supplementary information

HCT116-STR

HEK293T-STR

HT29-STR

Supplemental Figure legends

quantifications for Western blots

Western Blot images

supplyment1

supplymentary2

supplymentary3

supplymentary4

MS-oeHDAC6 specific proteins

aj-checklist

## Data Availability

The data that support the findings of our study are available from the corresponding author and supplementary information files.
