# Supplementary figures and images for: HDAC6-dependent deacetylation of TAK1 enhances sIL-6R release to promote macrophage M2 polarization in colon cancer
Source: Cell Death Dis. 2022 Oct 21;13(10):888. doi: 10.1038/s41419-022-05335-1 (PMC9587286; doi:10.1038/s41419-022-05335-1)

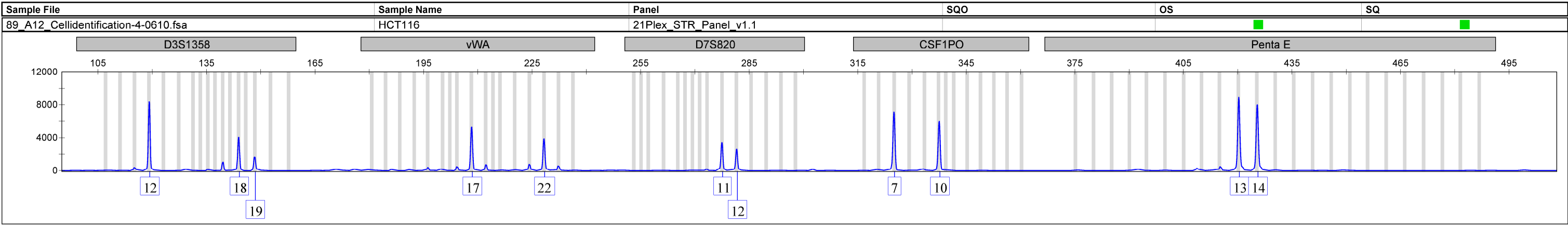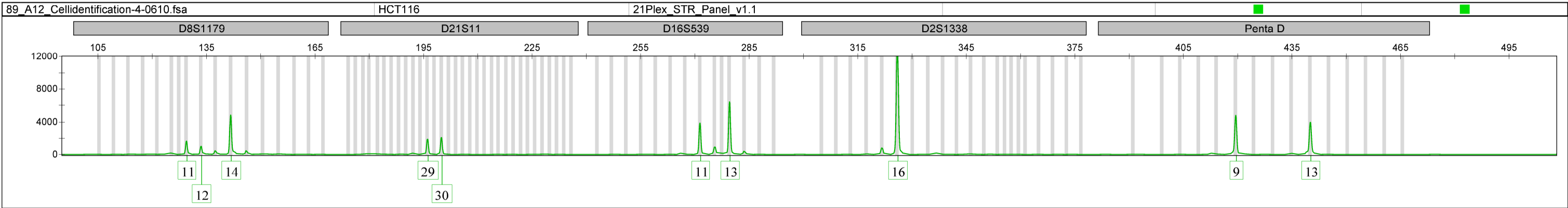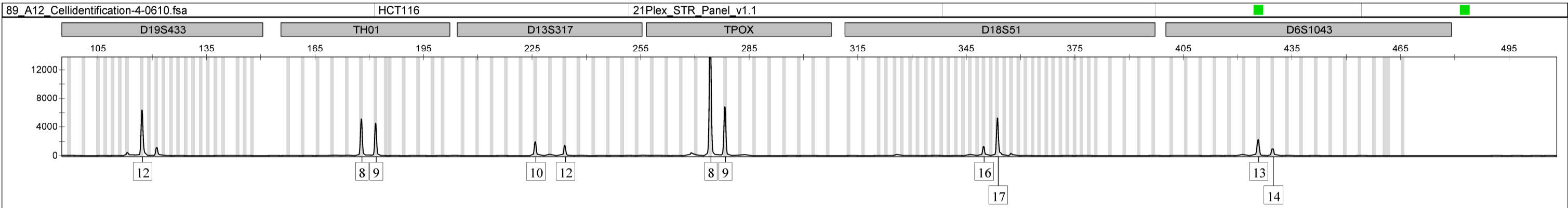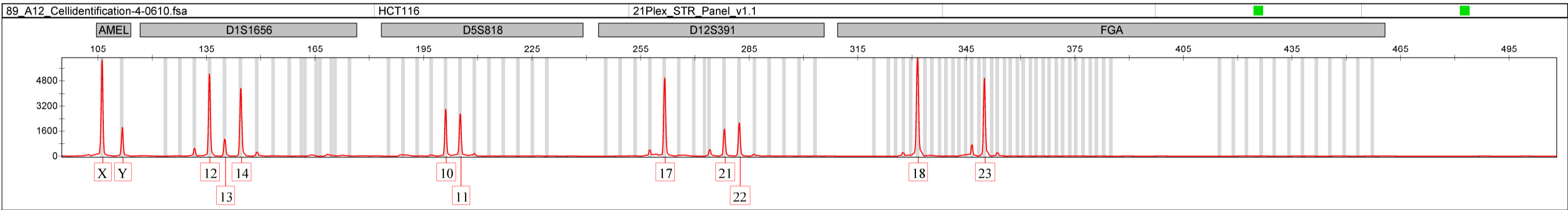

Supplement: Supplementary file 1 — HCT116-STR [file 41419_2022_5335_MOESM1_ESM.pdf]

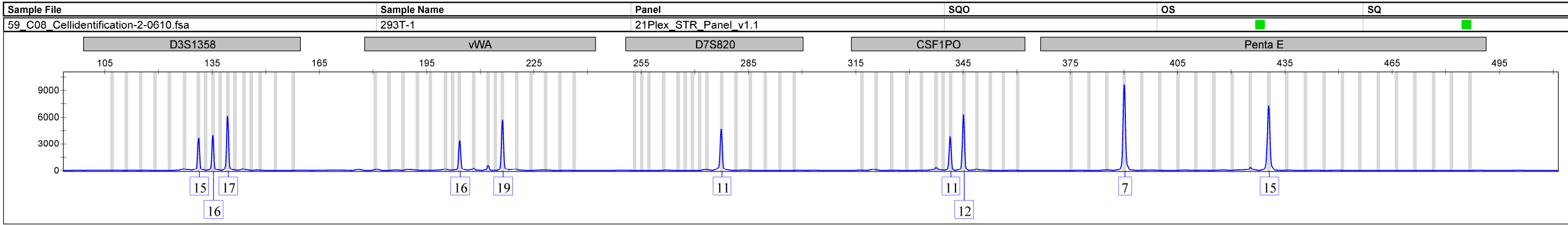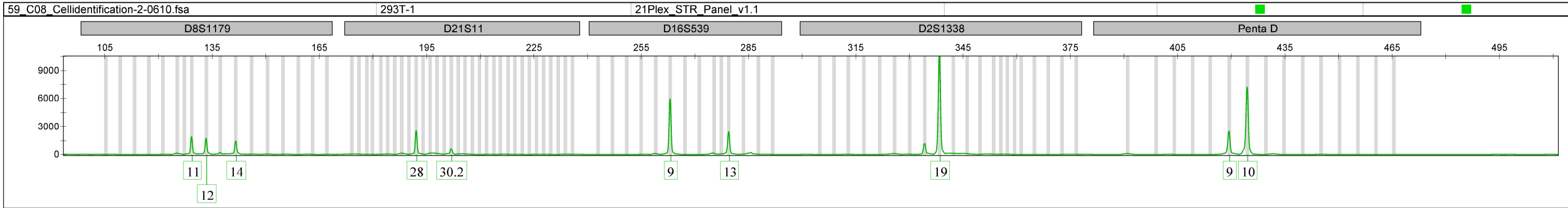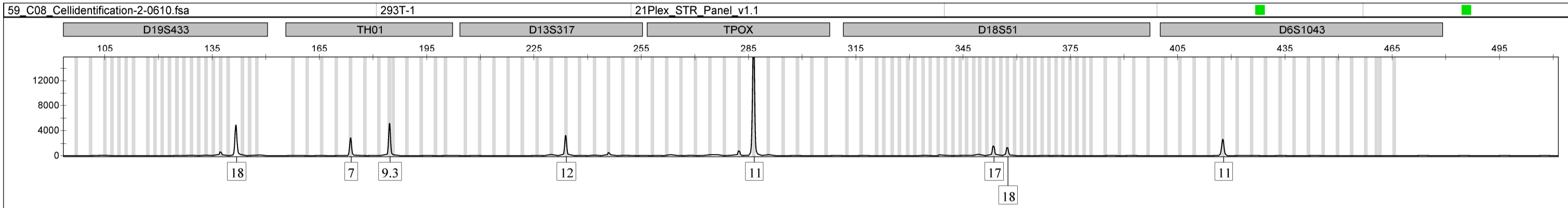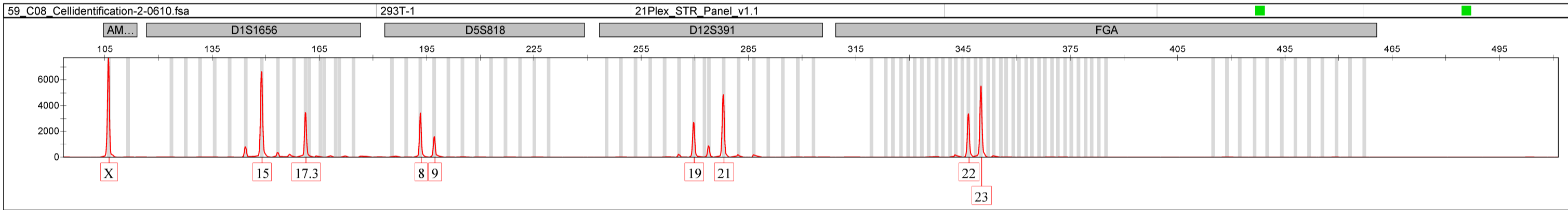

Supplement: Supplementary file 2 — HEK293T-STR [file 41419_2022_5335_MOESM2_ESM.pdf]

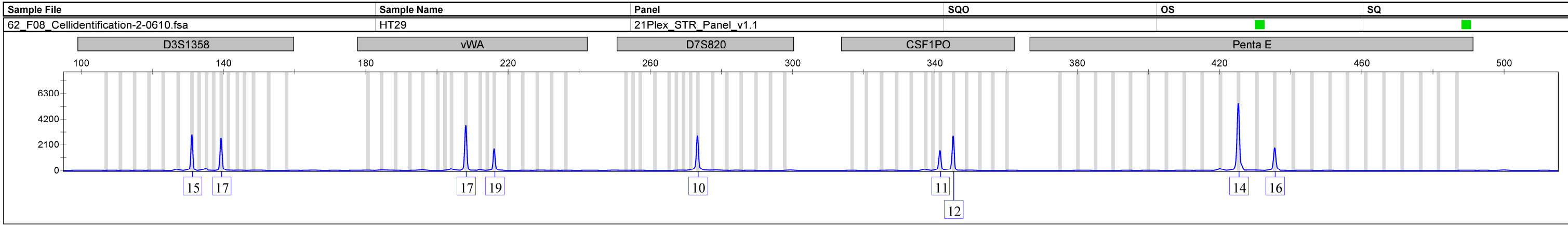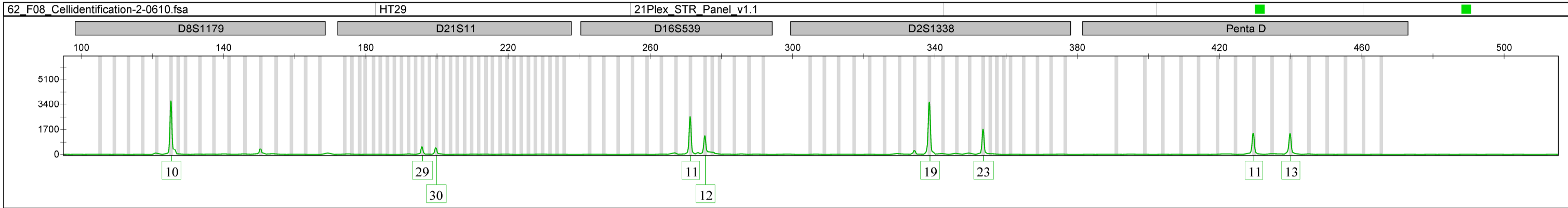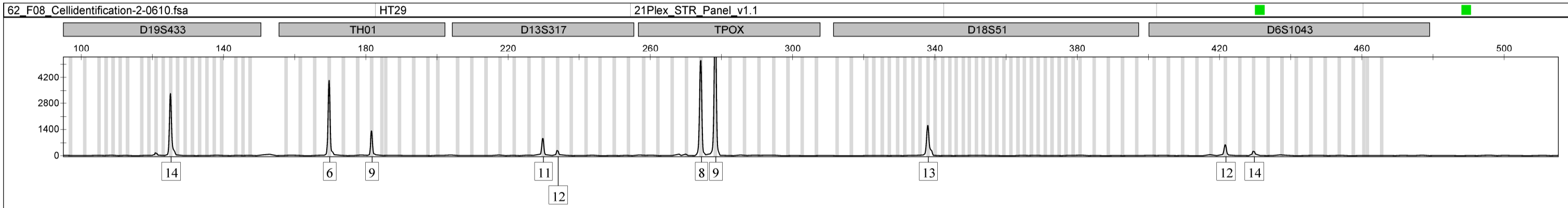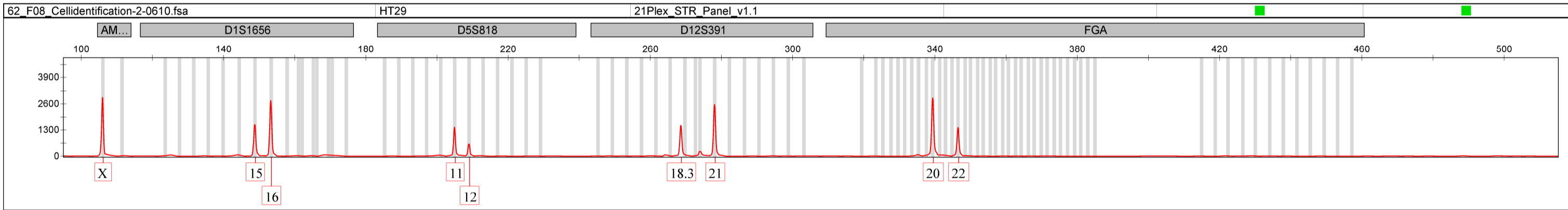

Supplement: Supplementary file 3 — HT29-STR [file 41419_2022_5335_MOESM3_ESM.pdf]

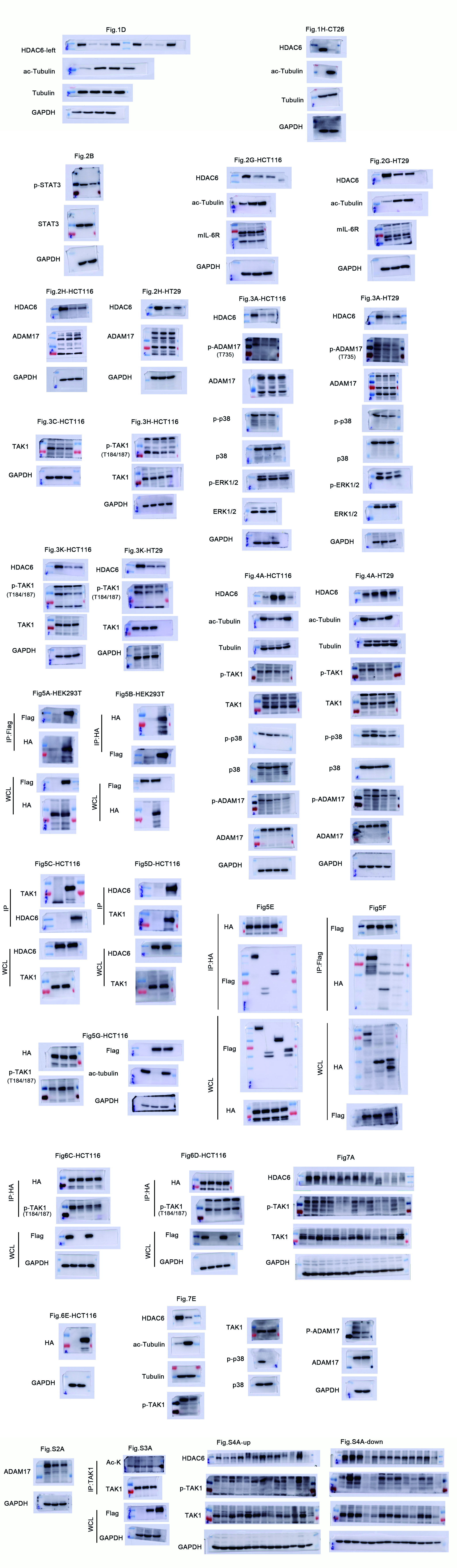

Supplement: Supplementary file 6 — Western Blot images [file 41419_2022_5335_MOESM6_ESM.tif]

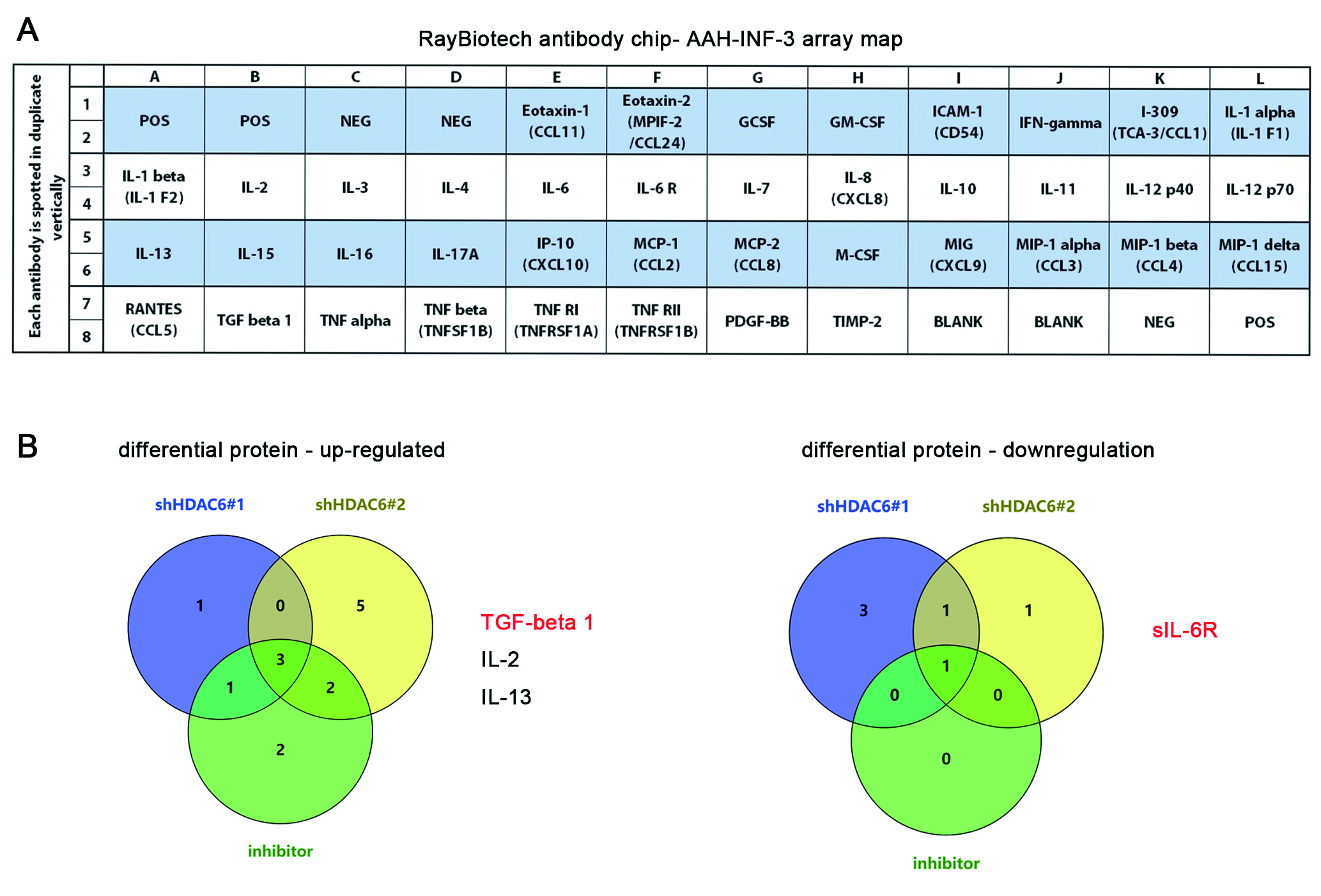

Supplement: Supplementary file 7 — supplyment1 [file 41419_2022_5335_MOESM7_ESM.tif]

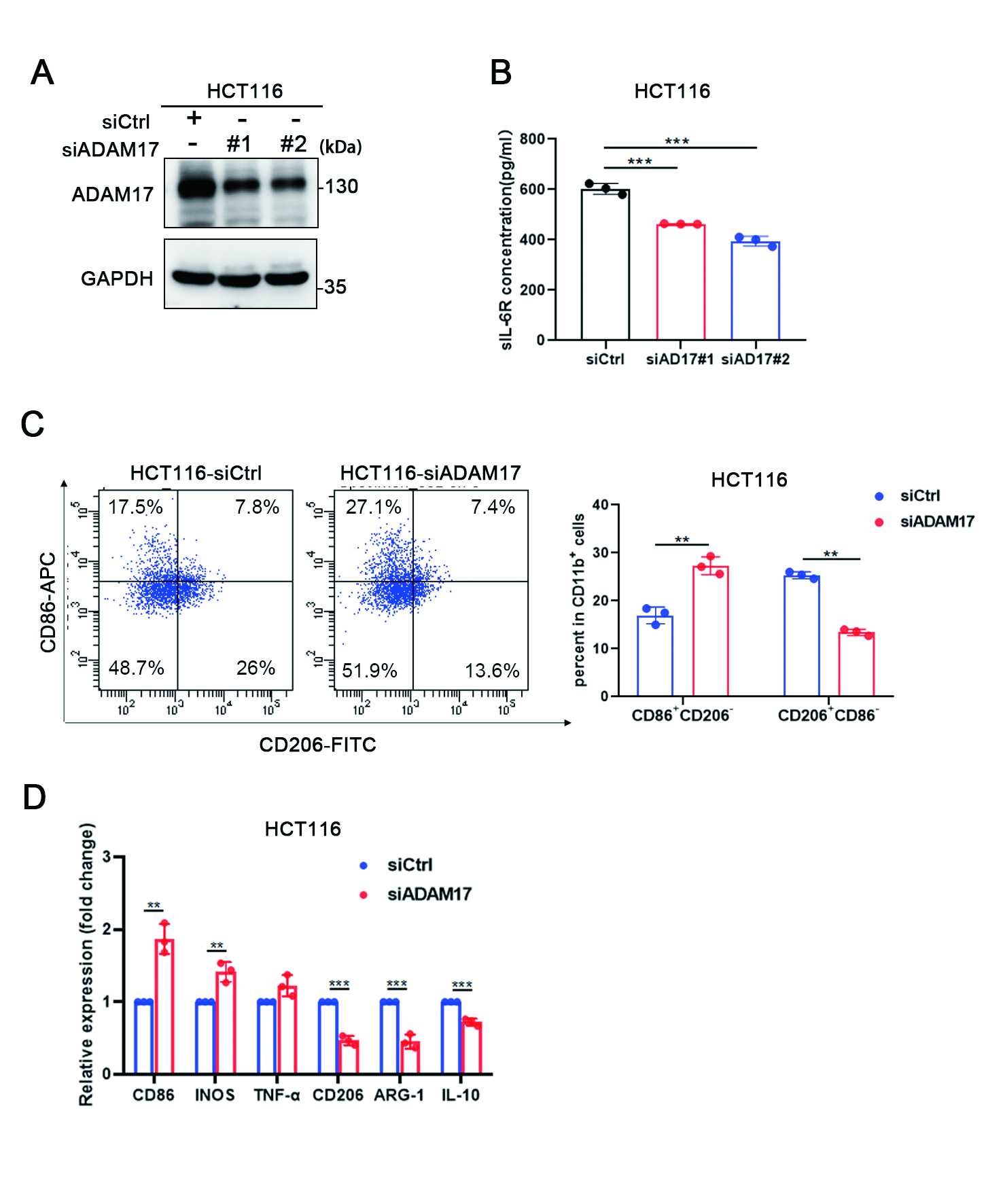

Supplement: Supplementary file 8 — supplymentary2 [file 41419_2022_5335_MOESM8_ESM.tif]

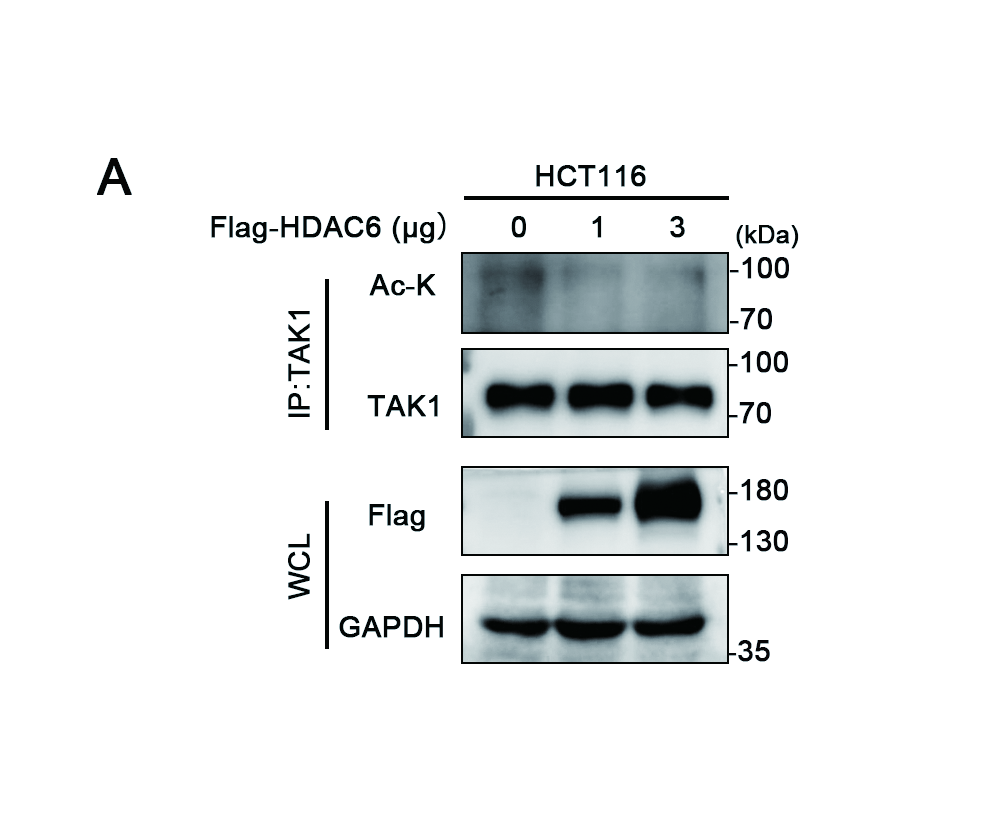

Supplement: Supplementary file 9 — supplymentary3 [file 41419_2022_5335_MOESM9_ESM.tif]

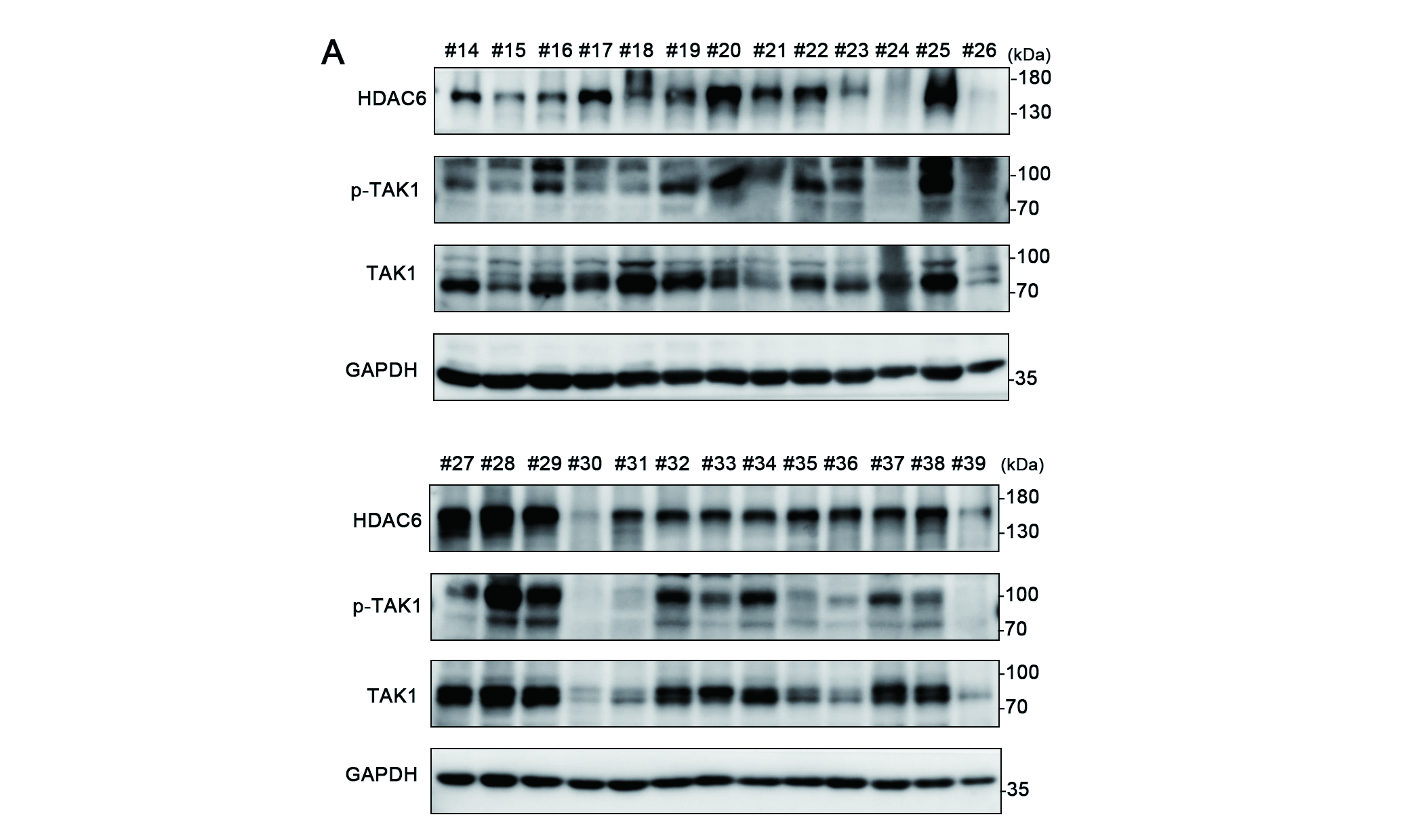

Supplement: Supplementary file 10 — supplymentary4 [file 41419_2022_5335_MOESM10_ESM.tif]
