# Supplementary material for: HDAC6-dependent deacetylation of TAK1 enhances sIL-6R release to promote macrophage M2 polarization in colon cancer
Source: Cell Death Dis. 2022 Oct 21;13(10):888. doi: 10.1038/s41419-022-05335-1 (PMC9587286; doi:10.1038/s41419-022-05335-1)
Supplement: Supplementary file 4 — Supplemental Figure legends [file 41419_2022_5335_MOESM4_ESM.docx]

**Supplemental Figure legends**

**Supplemental Fig 1**

**A.** Semiquantitative detection of 40 human inflammatory factors in colon cancer cell medium. **B.** Raw data obtained by chip scanning were processed by Raybiotech software to remove the chip background and normalize different chips. Differential proteins were screened according to a fold change ≤ 0.83 or ≥ 1.2. Selecting an average signal value > 150 for each group is recommended. The common upregulated genes were TGF-beta 1, IL-2, and IL-13; however, the average signal values of IL-2 and IL-13 were lower than 150. The common downregulated gene was sIL-6R.

**Supplemental Fig 2**

**A, B.** Transient knockdown of ADAM17 by siRNA in HCT116 cells and measurement of the sIL-6R content in the cell medium by ELISA. **C.** Flow cytometry assessed CD11b, CD86 and CD206 expression of co-cultured macrophages in siCtrl- and siADAM17-treated HCT116 cells. **D.** QPCR detected the expression of M1 and M2 polarization related genes of cocultured macrophages in siCtrl- and siADAM17-treated HCT116 cells. Data were shown as the mean ± SD, ** P < 0.01; *** P < 0.001.

**Supplemental Fig 3**

**A.** Acetylation of lysine residues in endogenous TAK1 measured after transfection with plasmids expressing increasing amounts of HDAC6 (µg). Whole-cell lysates were immunoprecipitated with an anti-TAK1 antibody, and the precipitated proteins were probed with an anti-acetylated lysine antibody. WCL is the abbreviation of whole cell lysate.

**Supplemental Fig 4**

**A.** Proteins were extracted from 38 fresh postoperative colon cancer tissues for western blot analysis of p-TAK1, TAK1, and HDAC6 protein levels. Relative protein levels were normalized by GAPDH. Twenty-five samples are shown.
